# Supplementary material for: A manganese(I) complex with a 190 ns metal-to-ligand charge transfer lifetime
Source: Nat Commun. 2025 Aug 22;16:7850. doi: 10.1038/s41467-025-63225-4 (PMC12373760; doi:10.1038/s41467-025-63225-4)
Supplement: Supplementary file 1 — Supplementary Information [file 41467_2025_63225_MOESM1_ESM.pdf]

A manganese(I) complex with a 190 ns metal-to-ligand charge transfer lifetime

Sandra Kronenberger, Robert Naumann, Christoph Förster, Nathan R. East, Jan Klett, and Katja Heinze*

Supplementary Information

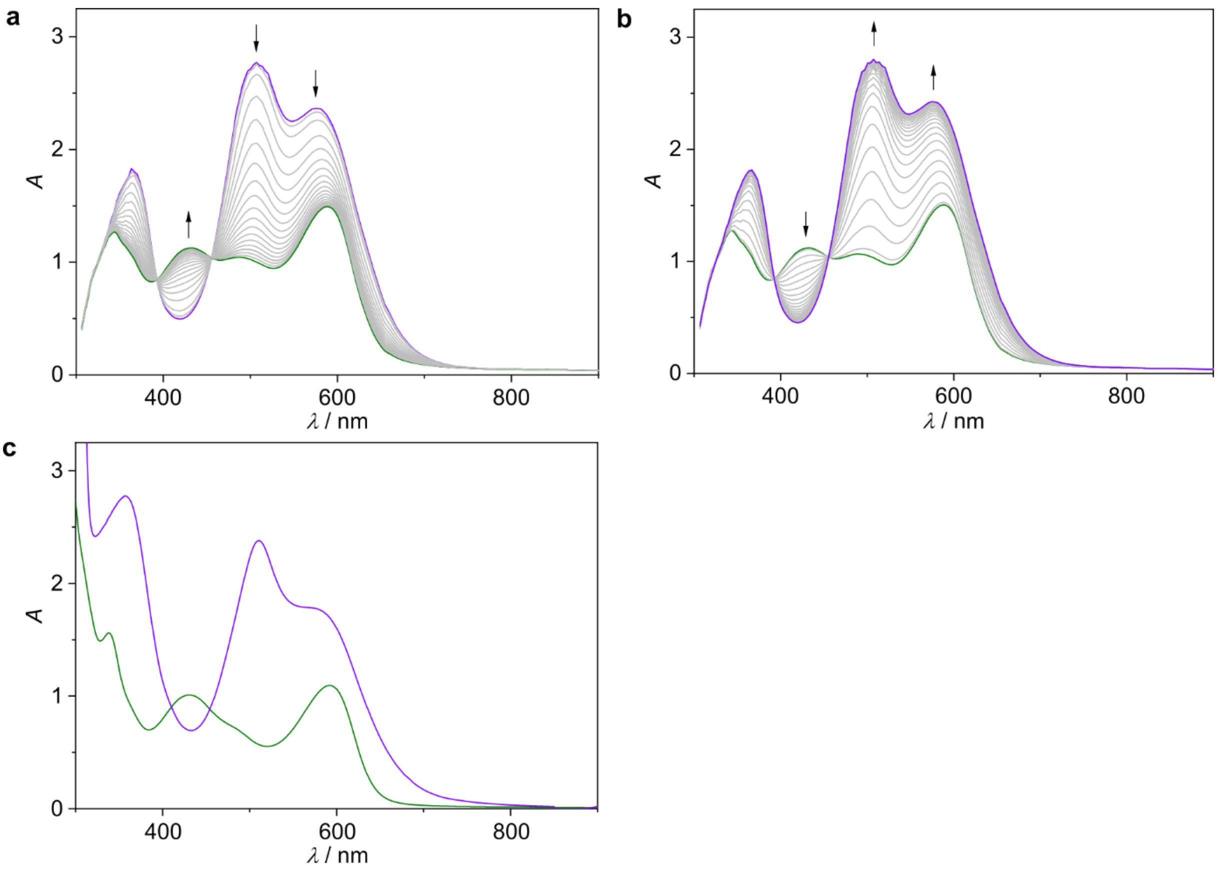

Supplementary Figure 1. UV-vis-NIR absorption spectra from spectroelectrochemical experiments of a solution of [Mn(pbmi)$_2$][OTf] (1 mM) in CH_3CN containing [^{n}Bu$_4$N][PF$_6$] (100 mM) as supporting electrolyte. **a,** UV-vis-NIR absorption spectra during electrochemical oxidation of [Mn(pbmi)$_2$]$^+$ to [Mn(pbmi)$_2$]$^{2+}$. **b,** UV-vis-NIR absorption spectra during subsequent electrochemical reduction of [Mn(pbmi)$_2$]$^{2+}$ to [Mn(pbmi)$_2$]$^+$. **c,** UV-vis-NIR absorption spectrum of [Mn(pbmi)$_2$]$^{2+}$ (green) in THF and UV-vis-NIR absorption spectrum of the solution after addition of pbmi (10 eq [H$_2$pbmi]Cl$_2$ and 20 eq Na[N(SiMe$_3$)$_2$]) at 195 K in THF resulting in the formation of the manganese(I) complex [Mn(pbmi)$_2$]$^+$ (purple).

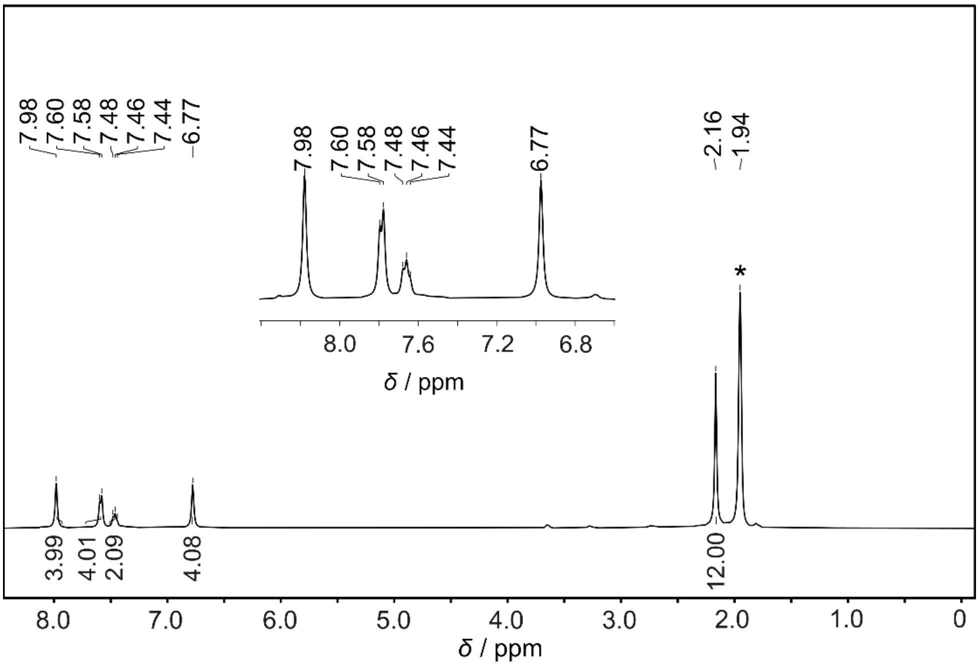

Supplementary Figure 2. ^{1}H NMR spectrum of [Mn(pbmi)$_2$][OTf] in CD_3CN at 293 K. The asterisk denotes residual solvent resonance.

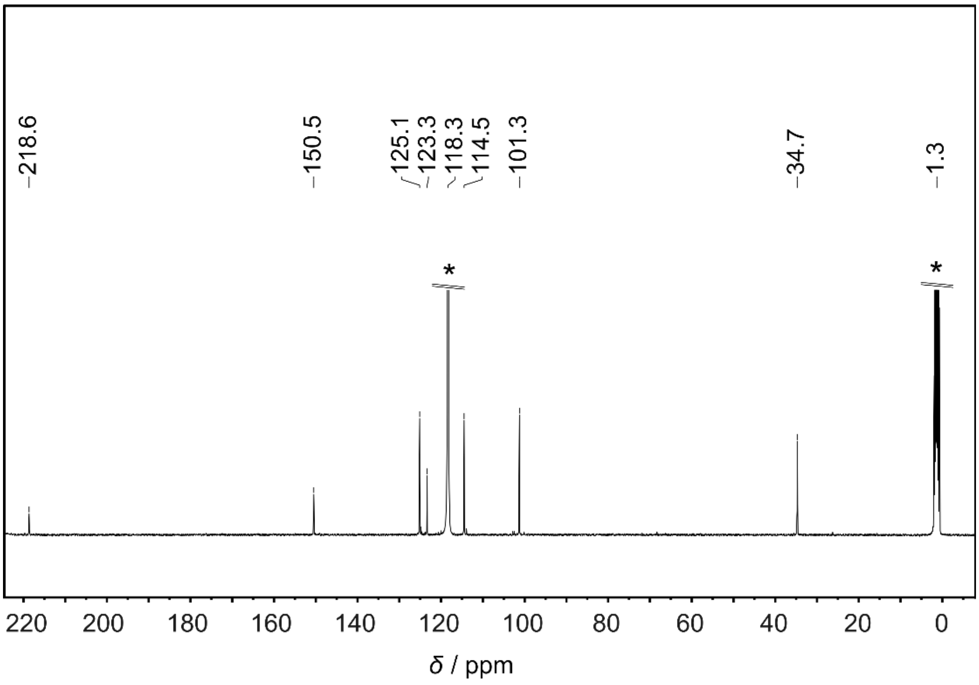

Supplementary Figure 3. ^{13}C{^{1}H} NMR spectrum of [Mn(pbmi)$_2$][OTf] in CD_3CN at 293 K. The asterisks denote solvent resonances.

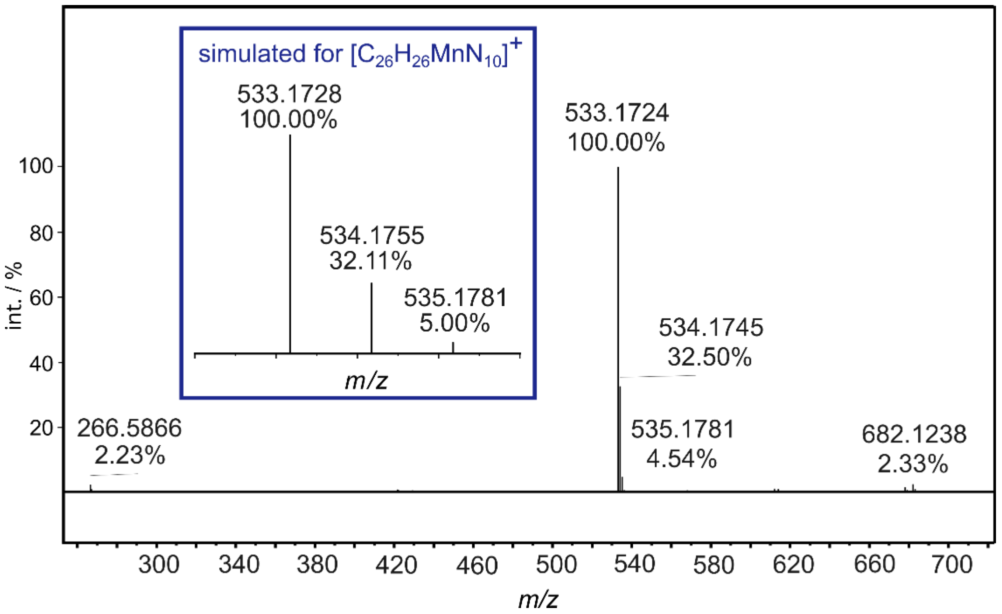

Supplementary Figure 4. ESI$^+$ mass spectrum of [Mn(pbmi)$_2$][OTf] in dry CH_3CN. The inset shows the calculated isotopic pattern for the major peak at *m*/*z* 533.1724 for [Mn(pbmi)$_2$]$^+$ ($C_{26}H_{26}MnN_6$).

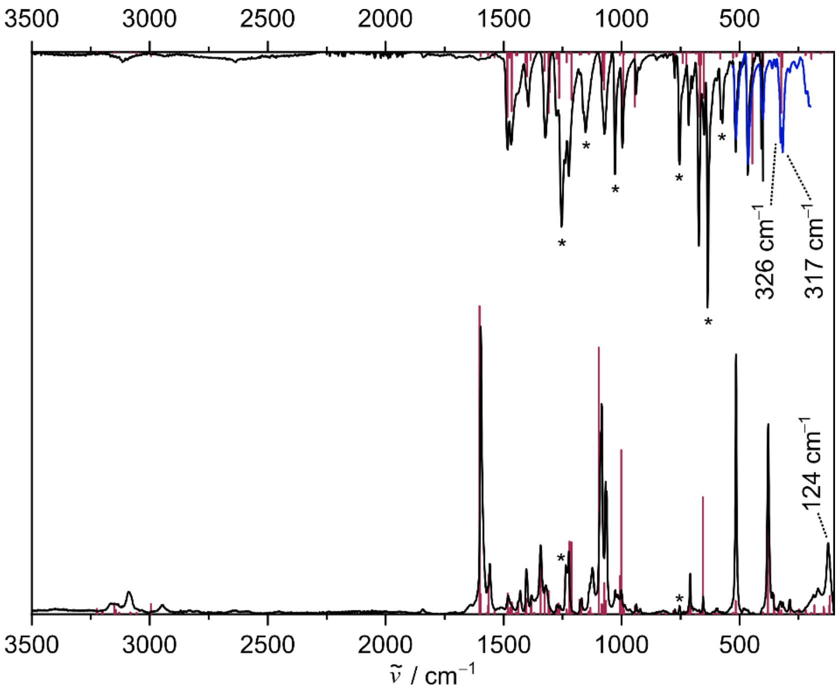

Supplementary Figure 5. Infrared (top) and Raman (bottom) spectrum of crystalline [Mn(pbmi)$_2$][OTf] at 293 K. The far-infrared spectrum is shown in blue. DFT calculated Raman and IR vibrational bands are scaled with a factor of 0.98 and are depicted as red vertical bars. Asterisks denote absorptions of the triflate counterion.

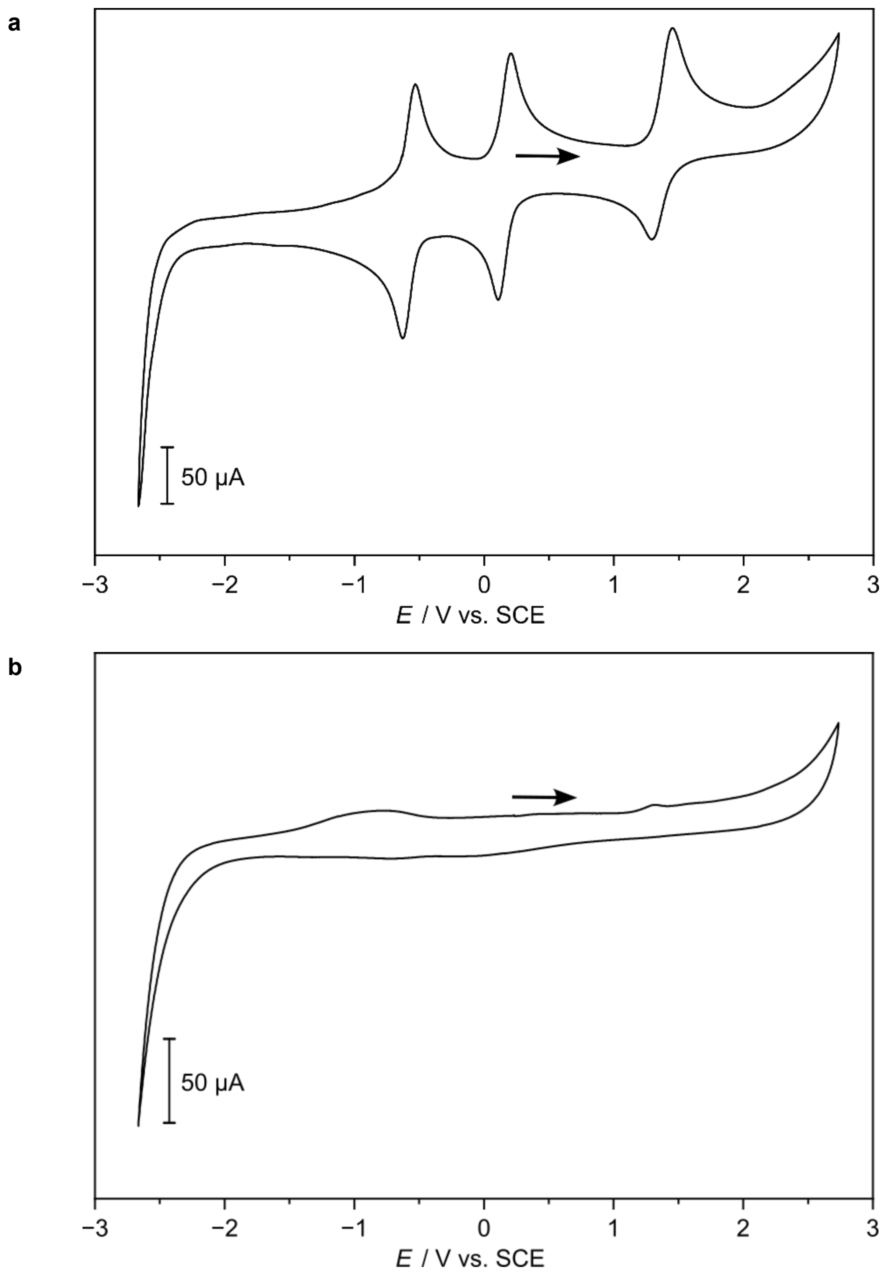

Supplementary Figure 6. a, Cyclic voltammogram of [Mn(pbmi)$_2$][OTf] (1 mM) in CH_3CN containing [^{n}Bu$_4$N][PF$_6$] (100 mM) as supporting electrolyte. **b,** Cyclic voltammogram of the electrolyte CH_3CN containing [^{n}Bu$_4$N][PF$_6$] (100 mM).

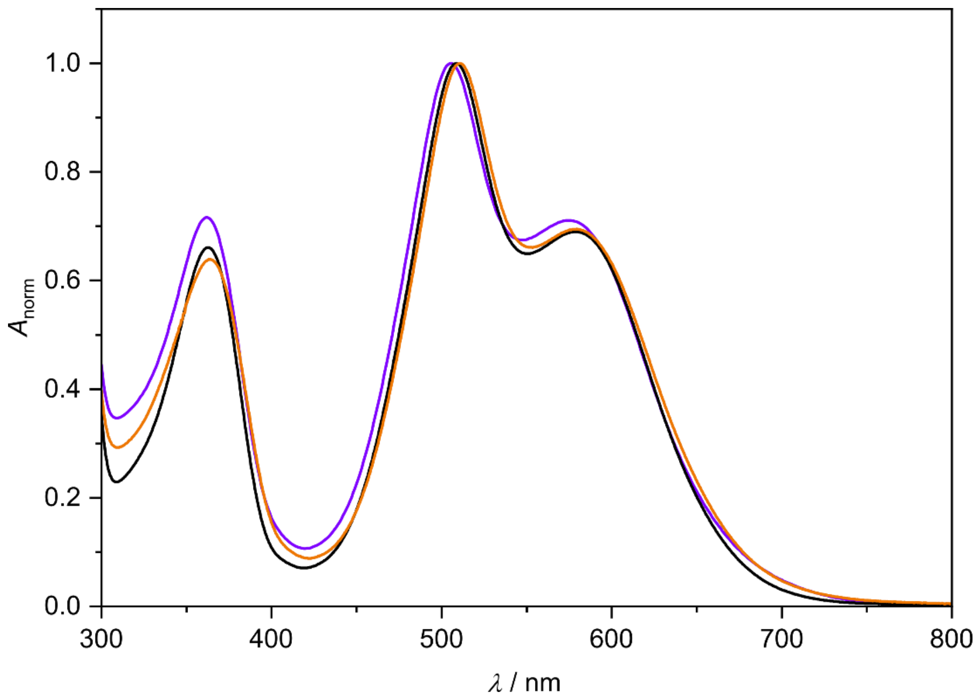

Supplementary Figure 7. Normalized absorption spectra of $[Mn(pbmi)_2][OTf]$ in CH_3CN (purple), THF (black) and CH_2Cl_2 (orange) at 293 K.

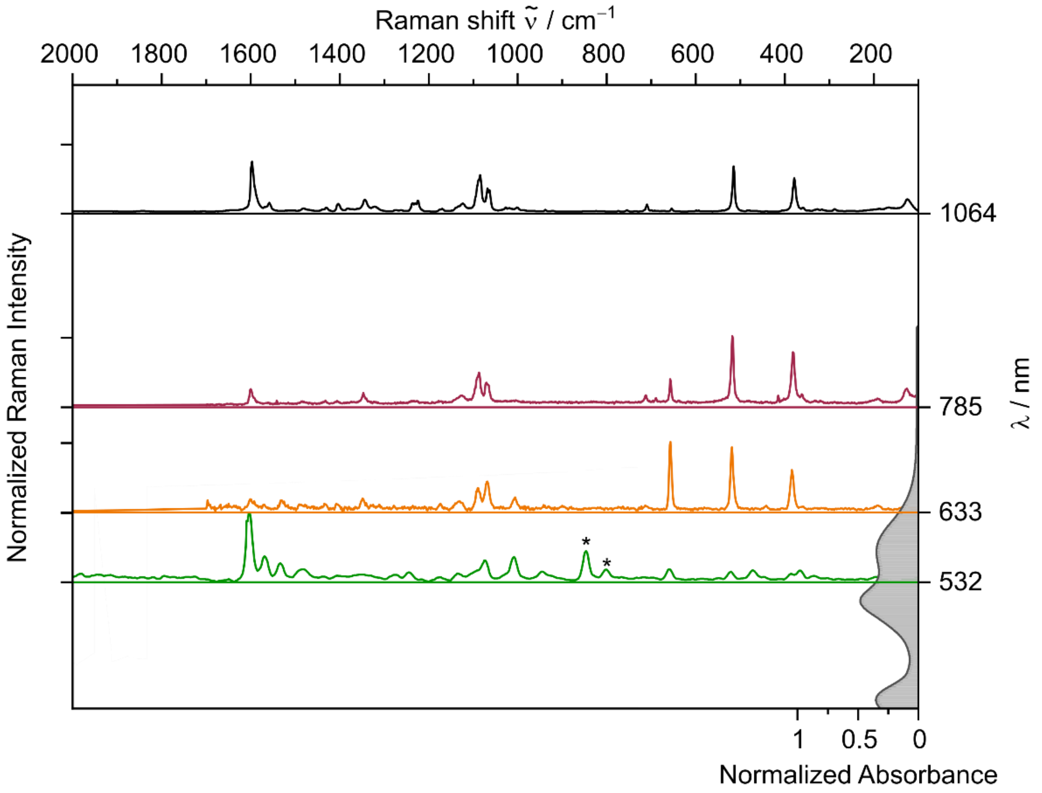

Supplementary Figure 8. Raman spectra of $[Mn(pbmi)_2][OTf]$ at 293 K with laser excitation at 532 nm (green, in d_8-THF; asterisks indicate solvent absorption bands), 633 nm (orange, solid), 785 nm (red, solid) and 1064 nm (black, solid). The absorption spectrum of $[Mn(pbmi)_2][OTf]$ in CH_3CN is given projected on the right y-axis for reference.

Supplementary Table 1. Charge transfer number analysis of the 50 lowest energy spin-allowed transitions calculated by Loewdin population analysis with the complex cation fragmented into manganese (Mn), pyridines (L) and NHCs (L'). Difference densities (red = electron loss; blue = electron gain; displayed at an isosurface value of 0.003 a.u.) of the 15 lowest spin-allowed transitions of $[Mn(pbmi)_2]^+$ with the respective calculated wavelengths (λ_{calcd}), wavelengths shifted by 0.33 eV to lower energies ($\lambda_{shifted}$), oscillator strengths (f) and dominant character.

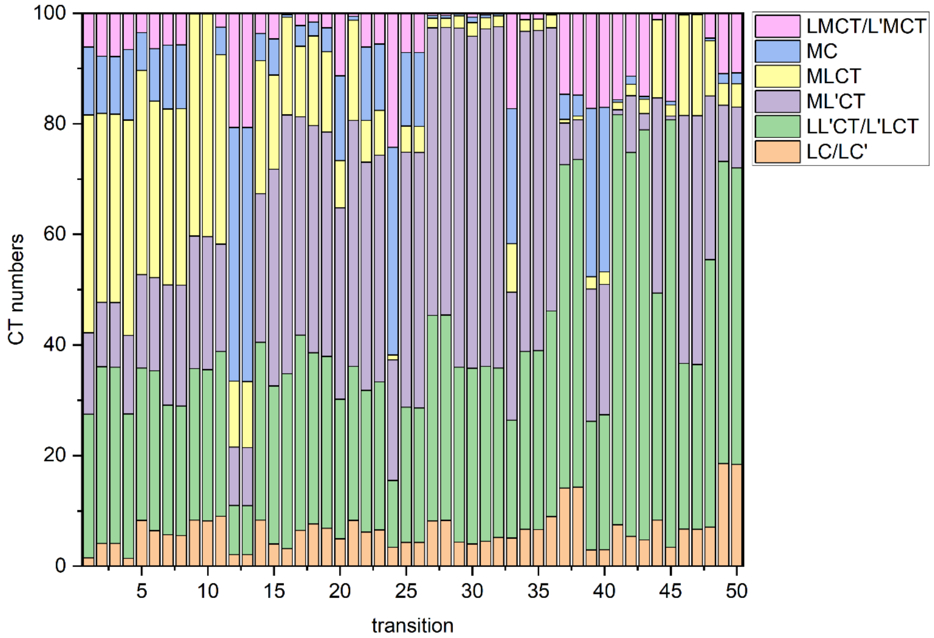

Number	TDDFT calculated difference density (isosurface value 0.003 a.u.)	λ_{calcd} / nm	$\lambda_{shifted}$ / nm (shifted by 0.33 eV to lower energy)	oscillator strength f	Character
1	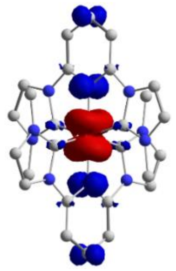	631.7	759.4	6.98E–06	MLCT d_{Mn}-p_{py} (39.4%)<br>ML'CT d_{Mn}-p_{NHC} (14.7%)<br>L'MCT/ LMCT p_L-d_{Mn} (6.12%)<br>L'LCT/LL'CT $p_{L'}$-p_L (26.0%)<br>L'C/LC p_L-p_L, $p_{L'}$-$p_{L'}$ (1.50%)<br>MC d_{Mn}-d_{Mn} (12.3%)
2	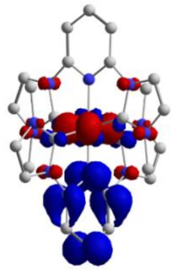	615.2	735.7	1.98E–03	MLCT d_{Mn}-p_{py} (34.1%)<br>ML'CT d_{Mn}-p_{NHC} (11.6%)<br>L'MCT/ LMCT p_L-d_{Mn} (7.79%)<br>L'LCT/LL'CT $p_{L'}$-p_L (32.0%)<br>L'C/LC p_L-p_L, $p_{L'}$-$p_{L'}$ (4.09%)<br>MC d_{Mn}-d_{Mn} (10.4%)
3	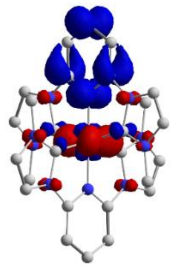	614.9	735.2	2.01E–03	MLCT d_{Mn}-p_{py} (34.1%)<br>ML'CT d_{Mn}-p_{NHC} (11.7%)<br>L'MCT/ LMCT p_L-d_{Mn} (7.85%)<br>L'LCT/LL'CT $p_{L'}$-p_L (32.0%)<br>L'C/LC p_L-p_L, $p_{L'}$-$p_{L'}$ (4.08%)<br>MC d_{Mn}-d_{Mn} (10.4%)

4	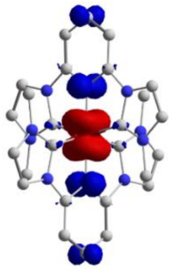	590.6	700.8	1.28E–06	MLCT d_{Mn}-p_{py} (38.9%)<br>ML'CT d_{Mn}-p_{NHC} (14.2%)<br>L'MCT/ LMCT p_{L}-d_{Mn} (6.60%)<br>L'LCT/LL'CT $p_{L'}$-p_{L} (26.1%)<br>L'C/LC p_{L}-p_{L}, $p_{L'}$-$p_{L'}$ (1.40%)<br>MC d_{Mn}-d_{Mn} (12.8%)
5	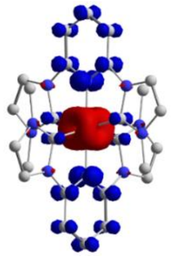	522.9	607.4	7.84E–02	MLCT d_{Mn}-p_{py} (37.0%)<br>ML'CT d_{Mn}-p_{NHC} (16.9%)<br>L'MCT/ LMCT p_{L}-d_{Mn} (3.50%)<br>L'LCT/LL'CT $p_{L'}$-p_{L} (27.6%)<br>L'C/LC p_{L}-p_{L}, $p_{L'}$-$p_{L'}$ (8.25%)<br>MC d_{Mn}-d_{Mn} (6.86%)
6	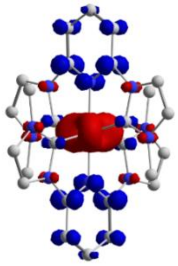	519.1	602.3	9.85E–06	MLCT d_{Mn}-p_{py} (31.9%)<br>ML'CT d_{Mn}-p_{NHC} (16.9%)<br>L'MCT/ LMCT p_{L}-d_{Mn} (6.46%)<br>L'LCT/LL'CT $p_{L'}$-p_{L} (28.9%)<br>L'C/LC p_{L}-p_{L}, $p_{L'}$-$p_{L'}$ (6.39%)<br>MC d_{Mn}-d_{Mn} (9.48%)
7	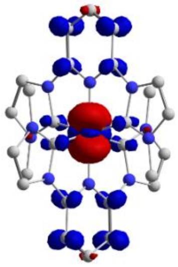	501.6	578.9	1.51E–02	MLCT d_{Mn}-p_{py} (31.8%)<br>ML'CT d_{Mn}-p_{NHC} (21.7%)<br>L'MCT/ LMCT p_{L}-d_{Mn} (5.81%)<br>L'LCT/LL'CT $p_{L'}$-p_{L} (23.4%)<br>L'C/LC p_{L}-p_{L}, $p_{L'}$-$p_{L'}$ (5.65%)<br>MC d_{Mn}-d_{Mn} (11.5%)
8	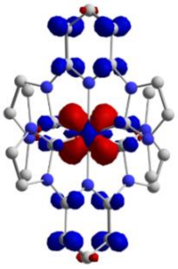	500.9	578.0	1.40E–02	MLCT d_{Mn}-p_{py} (31.9%)<br>ML'CT d_{Mn}-p_{NHC} (21.8%)<br>L'MCT/ LMCT p_{L}-d_{Mn} (5.76%)<br>L'LCT/LL'CT $p_{L'}$-p_{L} (23.5%)<br>L'C/LC p_{L}-p_{L}, $p_{L'}$-$p_{L'}$ (5.51%)<br>MC d_{Mn}-d_{Mn} (11.5%)
9	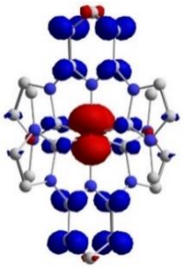	477.8	547.4	1.72E–02	MLCT d_{Mn}-p_{py} (40.2%)<br>ML'CT d_{Mn}-p_{NHC} (24.0%)<br>L'MCT/ LMCT p_{L}-d_{Mn} (1.12%)<br>L'LCT/LL'CT $p_{L'}$-p_{L} (27.4%)<br>L'C/LC p_{L}-p_{L}, $p_{L'}$-$p_{L'}$ (8.30%)<br>MC d_{Mn}-d_{Mn} (0.03%)

10	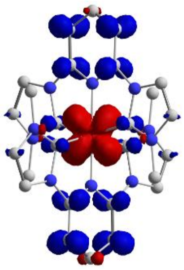	477.1	546.5	1.69E–02	MLCT d_{Mn}-p_{py} (40.3%)<br>ML'CT d_{Mn}-p_{NHC} (24.1%)<br>L'MCT/ LMCT p_{L}-d_{Mn} (1.10%)<br>L'LCT/LL'CT $p_{L'}$-p_{L} (27.4%)<br>L'C/LC p_{L}-p_{L}, $p_{L'}$-$p_{L'}$ (8.14%)<br>MC d_{Mn}-d_{Mn} (0.02%)
11	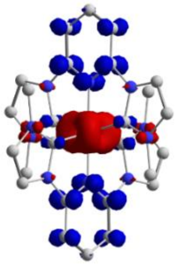	445.5	505.4	5.99E–01	MLCT d_{Mn}-p_{py} (34.2%)<br>ML'CT d_{Mn}-p_{NHC} (19.4%)<br>L'MCT/ LMCT p_{L}-d_{Mn} (2.55%)<br>L'LCT/LL'CT $p_{L'}$-p_{L} (29.8%)<br>L'C/LC p_{L}-p_{L}, $p_{L'}$-$p_{L'}$ (9.0%)<br>MC d_{Mn}-d_{Mn} (5.0%)
12	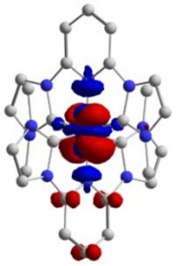	373.3	414.5	6.81E–04	MLCT d_{Mn}-p_{py} (11.9%)<br>ML'CT d_{Mn}-p_{NHC} (10.6%)<br>L'MCT/ LMCT p_{L}-d_{Mn} (20.7%)<br>L'LCT/LL'CT $p_{L'}$-p_{L} (8.89%)<br>L'C/LC p_{L}-p_{L}, $p_{L'}$-$p_{L'}$ (2.06%)<br>MC d_{Mn}-d_{Mn} (45.9%)
13	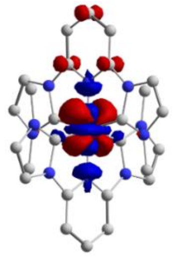	372.9	414.0	7.02E–04	MLCT d_{Mn}-p_{py} (11.9%)<br>ML'CT d_{Mn}-p_{NHC} (10.5%)<br>L'MCT/ LMCT p_{L}-d_{Mn} (20.7%)<br>L'LCT/LL'CT $p_{L'}$-p_{L} (8.85%)<br>L'C/LC p_{L}-p_{L}, $p_{L'}$-$p_{L'}$ (2.06%)<br>MC d_{Mn}-d_{Mn} (45.9%)
14	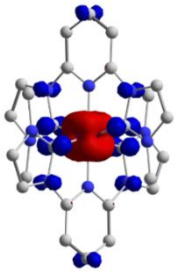	355.1	392.2	1.18E–06	MLCT d_{Mn}-p_{py} (24.1%)<br>ML'CT d_{Mn}-p_{NHC} (26.9%)<br>L'MCT/ LMCT p_{L}-d_{Mn} (3.66%)<br>L'LCT/LL'CT $p_{L'}$-p_{L} (32.1%)<br>L'C/LC p_{L}-p_{L}, $p_{L'}$-$p_{L'}$ (8.32%)<br>MC d_{Mn}-d_{Mn} (4.96%)
15	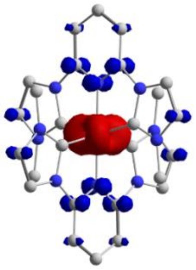	328.3	359.7	1.06E–05	MLCT d_{Mn}-p_{py} (17.1%)<br>ML'CT d_{Mn}-p_{NHC} (39.2%)<br>L'MCT/ LMCT p_{L}-d_{Mn} (4.64%)<br>L'LCT/LL'CT $p_{L'}$-p_{L} (28.6%)<br>L'C/LC p_{L}-p_{L}, $p_{L'}$-$p_{L'}$ (4.0%)<br>MC d_{Mn}-d_{Mn} (6.55%)

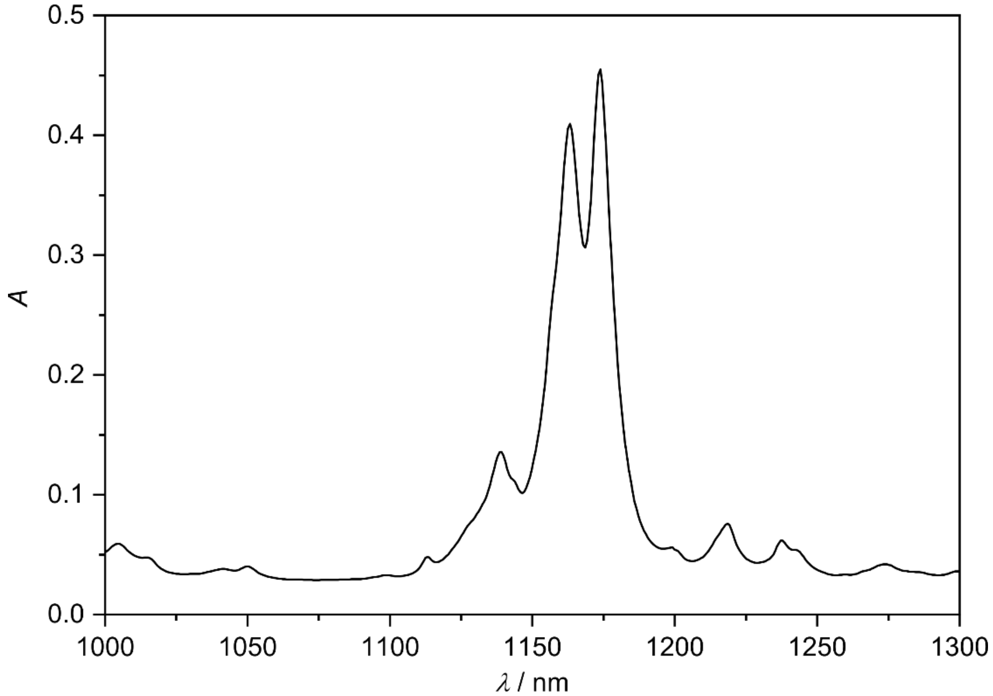

Supplementary Figure 9. NIR absorption spectrum of CH_3CN at 293 K showing the vibrational overtones that overlap with the phosphorescence emission of [Mn(pbmi)$_2$][OTf] causing re-absorption of the emitted photons.

Supplementary Table 2. SOC-TDDFT calculated energies E, oscillator strengths f (Supplementary Tables 3 – 6), phosphorescence rate constants k_p of the perturbed lowest triplet states at the ^{3}MLCT(1) geometry of $[Mn(pbmi)_2]^+$. The phosphorescence rate $k_p(T_i(\alpha))$ (in atomic units (a.u.)) of one spin sublevel $T_i(\alpha)$ ($\alpha = 1, 2, 3$) of state i can be identified with the oscillator strength $f_{S_0-T_i(\alpha)}$, the singlet-triplet energy difference, the refractive index of the solvent (CH_3CN, $n = 1.344$), according to the Strickler-Berg relationship (eq. (S1)).[1-3] After unit conversion of k_p from a.u. to s^{-1} with the a.u. of time $t = 2.418\ 10^{-17}$ s, the phosphorescence rates $k_p(T_i(\alpha))$ were obtained from the SOC-TDDFT calculated transition energies and oscillator strengths. Averaged phosphorescence rate constants $k_{p,av}$ were calculated according to eq. (S2) assuming a uniform population of all spin sublevels, according to a negligible zero-field splitting of 0.8, 1.3 and 18.6 cm^{-1} of the T_1, T_2 and T_3 sublevels from SOC-TDDFT.

**Triplet state (sublevel)**	**E / cm^{-1}**	**oscillator strength f**	**$k_p(T_i(\alpha))$ / s^{-1}**	**$k_{p,av}$ / s^{-1}**
$T_1(1)$	6075.4	0.000000750	33.4	
$T_1(2)$	6075.6	0.000001600	71.2	34.9
$T_1(3)$	6076.2	0.000000005	0.2	
$T_2(1)$	9934.7	0.000000031	3.7	
$T_2(2)$	9935.5	0.000000440	52.4	130.1
$T_2(3)$	9936.0	0.000002808	334.1	
$T_3(1)$	11688.7	0.000162309	26728.5	
$T_3(2)$	11693.9	0.000000617	101.7	8943.5
$T_3(3)$	11707.3	0.000000002	0.3	

$$k_p(T_i(\alpha)) = \frac{1}{\tau_p(T_i(\alpha))} = n^2 \frac{2 f_{S_0-T_i(\alpha)}}{c^3} \left(E(T_i(\alpha)) - E(S_0)\right)^2$$

in a.u., speed of light $c = 137.036$ a.u and refractive index n (eq. (S1))

$$k_{p,av} = \frac{k_p(T_i(1)) + k_p(T_i(2)) + k_p(T_i(3))}{3}$$ (eq. (S2))

Supplementary Table 3. TDDFT calculated absorption spectrum of $[Mn(pbmi)_2]^+$ via transition electric dipole moments.

State	Energy / cm^{-1}	λ / nm	oscillator strength f	T^2 / au^2	T_x / au	T_y / au	T_z / au
1	13658.7	732.1	0.001343239	0.03238	-0.17419	0.03854	0.02339
2	13668.9	731.6	0.000160961	0.00388	0.05674	-0.02487	-0.00618
3	15108.3	661.9	0.002328530	0.05074	0.02416	0.03630	0.22099
4	15614.0	640.5	0.000001110	0.00002	0.00089	-0.00463	0.00109
5	17642.4	566.8	0.167199580	3.11998	0.40426	1.69125	-0.31019
6	18168.0	550.4	0.010843454	0.19649	0.10159	0.42326	-0.08378
7	18949.2	527.7	0.020724526	0.36005	-0.05213	0.14986	0.57869
8	19044.8	525.1	0.011532265	0.19935	-0.41646	0.07026	-0.14483
9	20345.6	491.5	0.015123839	0.24472	0.48572	-0.09199	0.01825
10	20450.6	489.0	0.005664695	0.09119	0.07206	-0.10988	-0.27189
11	21029.3	475.5	0.313868853	4.91360	0.50029	2.12552	-0.38143
12	25007.3	399.9	0.000584748	0.00770	0.08489	-0.01896	-0.01151
13	25262.9	395.8	0.000135964	0.00177	0.00685	0.00190	0.04149
14	25446.9	393.0	0.000478041	0.00618	0.00480	-0.02901	0.07294
15	29374.2	340.4	0.000101776	0.00114	0.03255	-0.00841	0.00324
16	29977.1	333.6	0.000654092	0.00718	-0.05517	-0.05132	0.03881
17	30164.7	331.5	0.009510812	0.10380	0.29897	-0.07178	-0.09626
18	30206.4	331.1	0.048104987	0.52428	0.67080	-0.22827	-0.14902
19	30255.6	330.5	0.022112325	0.24060	-0.14321	-0.45999	0.09223
20	30302.7	330.0	0.016026749	0.17412	0.33087	-0.21936	-0.12853
21	30335.3	329.6	0.051429716	0.55814	0.71829	-0.12294	-0.16459
22	30442.0	328.5	0.007620080	0.08241	0.04214	0.26690	-0.09692
23	30636.1	326.4	0.093448746	1.00419	-0.16783	0.20519	-0.96640
24	30657.6	326.2	0.060457383	0.64921	0.26457	0.64984	0.39613
25	32687.3	305.9	0.001505398	0.01516	0.09783	-0.03848	-0.06412
26	32754.6	305.3	0.000106963	0.00108	0.01179	0.01652	0.02575
27	32913.3	303.8	0.043345735	0.43356	0.36959	-0.01036	0.54485
28	32950.3	303.5	0.036309201	0.36277	-0.45763	0.16465	0.35529
29	33027.8	302.8	0.004888032	0.04872	0.04568	0.21197	0.04128
30	33041.6	302.6	0.000080142	0.00080	-0.01347	-0.02402	-0.00634
31	33055.9	302.5	0.001832979	0.01826	0.12957	0.03730	-0.00872
32	33145.8	301.7	0.000906125	0.00900	0.00959	-0.09438	0.00038
33	33235.3	300.9	0.000215428	0.00213	0.04395	-0.01013	0.00999
34	34280.1	291.7	0.003255098	0.03126	0.14663	0.00859	0.09842
35	34338.3	291.2	0.005593247	0.05362	0.03296	0.22570	-0.03998
36	34393.5	290.8	0.001020121	0.00976	-0.02501	-0.00084	0.09560
37	34454.9	290.2	0.002581932	0.02467	0.01298	-0.01114	-0.15613
38	34519.5	289.7	0.000031853	0.00030	-0.01640	-0.00328	0.00489
39	35435.3	282.2	0.058129625	0.54005	-0.70593	0.18512	0.08630
40	35871.1	278.8	0.002189885	0.02010	-0.03032	-0.13752	0.01637
41	36555.5	273.6	0.083591339	0.75281	-0.11219	-0.11952	-0.85202
42	36763.8	272.0	0.011597109	0.10385	-0.07837	-0.31023	0.03825
43	37028.8	270.1	0.000009739	0.00009	0.00240	-0.00895	0.00079
44	37532.0	266.4	0.000000396	0.00000	0.00164	0.00040	0.00078
45	37884.2	264.0	0.000207081	0.00180	0.03698	-0.00961	-0.01843
46	37947.3	263.5	0.000162273	0.00141	-0.03629	0.00943	-0.00135
47	38090.6	262.5	0.000483936	0.00418	0.01713	0.06123	-0.01181
48	38173.5	262.0	0.070056871	0.60418	0.74700	-0.19872	-0.08173
49	38289.0	261.2	0.015523289	0.13347	0.09196	0.34686	-0.06859
50	38566.5	259.3	0.010873883	0.09282	0.06917	0.02903	0.29529
51	6067.4	1648.2	spin forbidden (mult=3)				
52	9929.3	1007.1	spin forbidden (mult=3)				
53	11701.0	854.6	spin forbidden (mult=3)				
54	11896.1	840.6	spin forbidden (mult=3)				
55	13104.1	763.1	spin forbidden (mult=3)				
56	14623.3	683.8	spin forbidden (mult=3)				
57	15814.9	632.3	spin forbidden (mult=3)				
58	17156.3	582.9	spin forbidden (mult=3)				
59	17376.5	575.5	spin forbidden (mult=3)				
60	19377.1	516.1	spin forbidden (mult=3)				
61	19663.1	508.6	spin forbidden (mult=3)				
62	20007.3	499.8	spin forbidden (mult=3)				
63	22777.0	439.0	spin forbidden (mult=3)				
64	23196.3	431.1	spin forbidden (mult=3)				
65	25522.6	391.8	spin forbidden (mult=3)				
66	27688.5	361.2	spin forbidden (mult=3)				
67	28580.9	349.9	spin forbidden (mult=3)				
68	28760.9	347.7	spin forbidden (mult=3)				
69	28958.7	345.3	spin forbidden (mult=3)				
70	29246.8	341.9	spin forbidden (mult=3)				
71	29603.6	337.8	spin forbidden (mult=3)				

72	29656.8	337.2	spin forbidden (mult=3)
73	29741.1	336.2	spin forbidden (mult=3)
74	30231.6	330.8	spin forbidden (mult=3)
75	30375.7	329.2	spin forbidden (mult=3)
76	30634.3	326.4	spin forbidden (mult=3)
77	30889.4	323.7	spin forbidden (mult=3)
78	31577.8	316.7	spin forbidden (mult=3)
79	31617.5	316.3	spin forbidden (mult=3)
80	31923.4	313.2	spin forbidden (mult=3)
81	32496.5	307.7	spin forbidden (mult=3)
82	32514.9	307.6	spin forbidden (mult=3)
83	32812.8	304.8	spin forbidden (mult=3)
84	32863.1	304.3	spin forbidden (mult=3)
85	33031.8	302.7	spin forbidden (mult=3)
86	33080.2	302.3	spin forbidden (mult=3)
87	33131.1	301.8	spin forbidden (mult=3)
88	33421.7	299.2	spin forbidden (mult=3)
89	33617.2	297.5	spin forbidden (mult=3)
90	33788.3	296.0	spin forbidden (mult=3)
91	34161.0	292.7	spin forbidden (mult=3)
92	34206.4	292.3	spin forbidden (mult=3)
93	34278.1	291.7	spin forbidden (mult=3)
94	35822.5	279.2	spin forbidden (mult=3)
95	36216.7	276.1	spin forbidden (mult=3)
96	36609.4	273.2	spin forbidden (mult=3)
97	36948.0	270.7	spin forbidden (mult=3)
98	37157.1	269.1	spin forbidden (mult=3)
99	37208.7	268.8	spin forbidden (mult=3)
100	37583.9	266.1	spin forbidden (mult=3)

Supplementary Table 4. SOC-TDDFT calculated absorption spectrum of $[Mn(pbmi)_2]^+$ of the 50 lowest energy states.

States	Energy / cm^{-1}	λ / nm	oscillator strength f	T^2 / au^2	T_x / au	T_y / au	T_z / au
0 1	6075.4	1646.0	0.000000750	0.00004	0.00057	0.00026	0.00634
0 2	6075.6	1645.9	0.000001600	0.00009	0.00893	0.00234	0.00120
0 3	6076.2	1645.8	0.000000005	0.00000	0.00032	0.00036	0.00011
0 4	9934.7	1006.6	0.000000031	0.00000	0.00046	0.00088	0.00023
0 5	9935.5	1006.5	0.000000440	0.00001	0.00369	0.00098	0.00016
0 6	9936.0	1006.4	0.000002808	0.00009	0.00112	0.00221	0.00932
0 7	11688.7	855.5	0.000162309	0.00457	0.01556	0.06474	0.01178
0 8	11693.9	855.1	0.000000617	0.00002	0.00076	0.00124	0.00391
0 9	11707.2	854.2	0.000000002	0.00000	0.00010	0.00022	0.00008
0 10	11902.1	840.2	0.000013224	0.00037	0.01841	0.00473	0.00210
0 11	11923.3	838.7	0.000168537	0.00465	0.01562	0.06539	0.01157
0 12	11924.3	838.6	0.000008686	0.00024	0.00122	0.00002	0.01544
0 13	13108.2	762.9	0.000059630	0.00150	0.00871	0.03711	0.00669
0 14	13108.9	762.8	0.000017296	0.00043	0.02005	0.00491	0.00289
0 15	13111.9	762.7	0.000000236	0.00001	0.00021	0.00240	0.00036
0 16	13676.0	731.2	0.001250321	0.03010	0.16821	0.03587	0.02276
0 17	13684.6	730.7	0.000285618	0.00687	0.07695	0.02951	0.00889
0 18	14617.3	684.1	0.000105862	0.00238	0.00549	0.00872	0.04773
0 19	14638.0	683.2	0.000080783	0.00182	0.00962	0.04085	0.00745
0 20	14640.3	683.0	0.000004756	0.00011	0.00999	0.00240	0.00121
0 21	15137.5	660.6	0.002313759	0.05032	0.02404	0.03605	0.22010
0 22	15630.4	639.8	0.000001013	0.00002	0.00089	0.00441	0.00105
0 23	15815.2	632.3	0.000000002	0.00000	0.00015	0.00013	0.00005
0 24	15818.2	632.2	0.000015645	0.00033	0.01745	0.00446	0.00116
0 25	15820.0	632.1	0.000009671	0.00020	0.00060	0.00192	0.01404
0 26	17138.3	583.5	0.000190261	0.00365	0.01377	0.05805	0.00978
0 27	17154.5	582.9	0.000001376	0.00003	0.00110	0.00493	0.00094
0 28	17168.5	582.5	0.000019057	0.00037	0.01877	0.00362	0.00020
0 29	17386.6	575.2	0.000069542	0.00132	0.00187	0.00875	0.03517
0 30	17397.8	574.8	0.003800574	0.07192	0.06145	0.25701	0.04568
0 31	17409.4	574.4	0.000011280	0.00021	0.00278	0.01411	0.00253
0 32	17664.1	566.1	0.162370048	3.02615	0.39814	1.66564	0.30543
0 33	18189.4	549.8	0.009633227	0.17435	0.09569	0.39868	0.07909
0 34	18964.9	527.3	0.020582787	0.35730	0.04842	0.14783	0.57715
0 35	19062.6	524.6	0.011418617	0.19720	0.41574	0.07144	0.13876
0 36	19350.9	516.8	0.000000019	0.00000	0.00043	0.00014	0.00035
0 37	19357.7	516.6	0.000008141	0.00014	0.00503	0.00278	0.01027
0 38	19367.3	516.3	0.000162224	0.00276	0.05035	0.01445	0.00367
0 39	19654.7	508.8	0.002502125	0.04191	0.04749	0.19628	0.03362
0 40	19691.4	507.8	0.000000012	0.00000	0.00025	0.00010	0.00035
0 41	19696.0	507.7	0.000002698	0.00005	0.00178	0.00167	0.00626
0 42	20015.1	499.6	0.000599235	0.00986	0.09652	0.02282	0.00436
0 43	20032.4	499.2	0.001793031	0.02947	0.03964	0.16474	0.02752
0 44	20043.0	498.9	0.000000077	0.00000	0.00057	0.00095	0.00019
0 45	20383.3	490.6	0.014318684	0.23126	0.47221	0.08796	0.02330
0 46	20477.1	488.4	0.005682738	0.09136	0.07832	0.10923	0.27073
0 47	21050.6	475.0	0.319895250	5.00287	0.50480	2.14474	0.38489
0 48	22770.7	439.2	0.000308571	0.00446	0.01520	0.06409	0.01108
0 49	22771.4	439.1	0.000001320	0.00002	0.00149	0.00406	0.00065
0 50	22784.3	438.9	0.000001199	0.00002	0.00078	0.00010	0.00409

Supplementary Table 5. Eigenvectors of the SOC matrix (threshold 0.0100) of the 50 lowest energy states.

State	Energy / cm^{-1}	Weight	Real	Imag	Root	Spin	Ms
STATE 0:	0.00						
		0.99948	-0.97737	0.21031 :	0	0	0
STATE 1:	6075.36						
		0.49709	-0.16730	-0.68491 :	1	1	-1
		0.49709	-0.17310	0.68346 :	1	1	1
STATE 2:	6075.62						
		0.96867	0.92737	-0.32965 :	1	1	0
		0.01547	0.10532	0.06616 :	1	1	-1
		0.01547	-0.03993	0.11779 :	1	1	1
STATE 3:	6076.16						
		0.02555	-0.15543	0.03736 :	1	1	0
		0.48705	0.31468	0.62292 :	1	1	-1
		0.48705	0.00278	0.69789 :	1	1	1
STATE 4:	9934.74						
		0.49802	-0.34953	-0.61307 :	2	1	-1
		0.49802	-0.03578	-0.70480 :	2	1	1
STATE 5:	9935.47						
		0.98996	0.96647	-0.23642 :	2	1	0
STATE 6:	9936.01						
		0.49625	0.59317	0.38000 :	2	1	-1
		0.49625	-0.35357	-0.60929 :	2	1	1
STATE 7:	11688.66						
		0.87987	0.21364	0.91336 :	3	1	0
		0.01721	-0.07639	0.10663 :	3	1	-1
		0.04109	-0.18163	0.08997 :	4	1	-1
		0.01721	-0.11576	-0.06169 :	3	1	1
		0.04109	-0.20269	-0.00007 :	4	1	1
STATE 8:	11693.95						
		0.02731	0.03830	-0.16075 :	3	1	0
		0.07307	0.06265	-0.26295 :	4	1	0
		0.44783	-0.66920	-0.00221 :	3	1	-1
		0.44783	-0.59829	-0.29979 :	3	1	1
STATE 9:	11707.25						
		0.49431	-0.33482	-0.61823 :	3	1	-1
		0.49431	0.00250	0.70306 :	3	1	1
STATE 10:	11902.12						
		0.49348	0.64270	-0.28358 :	4	1	-1
		0.49348	-0.70026	-0.05582 :	4	1	1
STATE 11:	11923.29						
		0.08106	0.07337	0.27509 :	3	1	0
		0.03368	0.04730	0.17733 :	4	1	0
		0.44032	0.58557	-0.31212 :	4	1	-1
		0.44032	0.66323	-0.02096 :	4	1	1
STATE 12:	11924.28						
		0.88064	-0.76791	0.53940 :	4	1	0
		0.03664	-0.14258	-0.12770 :	3	1	-1
		0.02110	-0.14033	-0.03752 :	4	1	-1
		0.03664	-0.07176	-0.17744 :	3	1	1
		0.02110	0.01231	-0.14474 :	4	1	1
STATE 13:	13108.17						
		0.49572	-0.32424	-0.62496 :	5	1	-1
		0.49572	-0.00505	0.70405 :	5	1	1
STATE 14:	13108.90						
		0.01623	0.12444	-0.02728 :	5	1	0
		0.48740	-0.30363	-0.62865 :	5	1	-1
		0.48740	0.01283	-0.69802 :	5	1	1
STATE 15:	13111.87						
		0.97660	-0.18488	-0.97078 :	5	1	0
		0.01044	0.06264	-0.08071 :	5	1	-1
		0.01044	0.08792	0.05204 :	5	1	1
STATE 16:	13676.05						
		0.97905	-0.16726	-0.97523 :	1	0	0
		0.01231	-0.01875	-0.10933 :	2	0	0
STATE 17:	13684.58						
		0.01171	0.03595	0.10205 :	1	0	0
		0.98405	-0.32961	-0.93563 :	2	0	0
STATE 18:	14617.33						
		0.03547	0.04257	-0.18347 :	3	0	0
		0.92653	0.93766	0.21756 :	6	1	0
		0.01825	0.06407	0.11895 :	6	1	-1
		0.01825	-0.10990	0.07858 :	6	1	1

```
STATE 19:  14638.00
                   0.02969      0.00712      0.17215 : 6     1     0
                   0.48334      0.66916     -0.18859 : 6     1    -1
                   0.48334      0.68245      0.13267 : 6     1     1
STATE 20:  14640.35
                   0.49378     -0.12212     -0.69200 : 6     1    -1
                   0.49378     -0.22355      0.66619 : 6     1     1
STATE 21:  15137.55
                   0.96053     -0.89580      0.39758 : 3     0     0
                   0.03464      0.07550      0.17011 : 6     1     0
STATE 22:  15630.38
                   0.99574      0.98803      0.13980 : 4     0     0
STATE 23:  15815.20
                   0.03103      0.17519     -0.01824 : 7     1     0
                   0.48012     -0.22873     -0.65407 : 7     1    -1
                   0.48012      0.08908     -0.68716 : 7     1     1
STATE 24:  15818.23
                   0.93968      0.93414     -0.25896 : 7     1     0
                   0.02728      0.15579      0.05488 : 7     1    -1
                   0.02728     -0.10530      0.12726 : 7     1     1
STATE 25:  15820.02
                   0.02347     -0.11428     -0.10203 : 7     1     0
                   0.48449      0.62116      0.31409 : 7     1    -1
                   0.48449     -0.38221     -0.58173 : 7     1     1
STATE 26:  17138.25
                   0.03455     -0.04243     -0.18096 : 8     1     0
                   0.08298      0.06575      0.28046 : 9     1     0
                   0.43356      0.31638      0.57747 : 8     1    -1
                   0.43356      0.02675     -0.65791 : 8     1     1
STATE 27:  17154.52
                   0.86251      0.50120      0.78186 : 8     1     0
                   0.03117      0.01873      0.17555 : 8     1    -1
                   0.03294     -0.13523     -0.12104 : 9     1    -1
                   0.03117     -0.15170     -0.09031 : 8     1     1
                   0.03294      0.05353      0.17342 : 9     1     1
STATE 28:  17168.46
                   0.02893      0.16312     -0.04823 : 8     1     0
                   0.48213     -0.34730     -0.60126 : 8     1    -1
                   0.48213     -0.03552     -0.69345 : 8     1     1
STATE 29:  17386.58
                   0.02284      0.15100     -0.00647 : 9     1     0
                   0.48516     -0.19268     -0.66936 : 9     1    -1
                   0.48516      0.13472     -0.68338 : 9     1     1
STATE 30:  17397.81
                   0.03018      0.16327     -0.05937 : 5     0     0
                   0.84392      0.31392      0.86335 : 9     1     0
                   0.04593     -0.12215     -0.17608 : 8     1    -1
                   0.01488     -0.03986      0.11529 : 9     1    -1
                   0.04593      0.01948      0.21342 : 8     1     1
                   0.01488     -0.10460     -0.06277 : 9     1     1
STATE 31:  17409.42
                   0.06750      0.05852      0.25313 : 8     1     0
                   0.01150     -0.02416     -0.10448 : 9     1     0
                   0.45784      0.31353      0.59962 : 9     1    -1
                   0.45784      0.01852     -0.67639 : 9     1     1
STATE 32:  17664.08
                   0.96585      0.95103     -0.24778 : 5     0     0
                   0.03059     -0.04410     -0.16926 : 9     1     0
STATE 33:  18189.36
                   0.98935     -0.98327      0.15012 : 6     0     0
STATE 34:  18964.89
                   0.98906      0.25846     -0.96034 : 7     0     0
STATE 35:  19062.58
                   0.99494      0.96184      0.26422 : 8     0     0
STATE 36:  19350.87
                   0.02840     -0.05402      0.15962 : 10     1     0
                   0.02263      0.04822     -0.14249 : 12     1     0
                   0.43474      0.65732      0.05166 : 10     1    -1
                   0.03904      0.01460     -0.19705 : 11     1    -1
                   0.43474      0.55360      0.35814 : 10     1     1
                   0.03904     -0.10806      0.16542 : 11     1     1
STATE 37:  19357.66
                   0.45635      0.45759      0.49695 : 10     1    -1
                   0.03496      0.13816     -0.12600 : 11     1    -1
                   0.45635     -0.15909     -0.65654 : 10     1     1
                   0.03496      0.18151     -0.04491 : 11     1     1
STATE 38:  19367.34
                   0.93754      0.93797     -0.24033 : 10     1     0
```

```
                 0.01574      0.07766      0.09853 : 10     1    -1
                 0.01002     -0.04758     -0.08807 : 12     1    -1
                 0.01574     -0.02071      0.12374 : 10     1     1
                 0.01002     -0.00064     -0.10010 : 12     1     1
STATE 39:  19654.72
                 0.91840     -0.13537      0.94872 : 11     1     0
                 0.01444     -0.11706      0.02721 : 11     1    -1
                 0.02340     -0.01703     -0.15203 : 12     1    -1
                 0.01444     -0.10478     -0.05886 : 11     1     1
                 0.02340     -0.05887      0.14120 : 12     1     1
STATE 40:  19691.35
                 0.01882     -0.01178     -0.13670 : 12     1     0
                 0.04390     -0.19813      0.06813 : 10     1    -1
                 0.44460     -0.21945     -0.62964 : 11     1    -1
                 0.04390     -0.20687     -0.03322 : 10     1     1
                 0.44460     -0.10847      0.65790 : 11     1     1
STATE 41:  19696.04
                 0.02734     -0.05614     -0.15553 : 11     1     0
                 0.03901      0.11081      0.16351 : 10     1    -1
                 0.44364     -0.55267      0.37175 : 11     1    -1
                 0.03901     -0.01917     -0.19659 : 10     1     1
                 0.44364     -0.66269      0.06693 : 11     1     1
STATE 42:  20015.09
                 0.04174      0.06657      0.19314 : 9     0     0
                 0.02545      0.15082     -0.05198 : 10     1     0
                 0.03016     -0.16419      0.05659 : 12     1     0
                 0.45008      0.36120      0.56534 : 12     1    -1
                 0.45008      0.06384      0.66783 : 12     1     1
STATE 43:  20032.39
                 0.04718     -0.04124     -0.21326 : 11     1     0
                 0.47280     -0.28850     -0.62415 : 12     1    -1
                 0.47280     -0.03500      0.68671 : 12     1     1
STATE 44:  20043.03
                 0.92456      0.22375     -0.93515 : 12     1     0
                 0.01588      0.00249      0.12599 : 11     1    -1
                 0.01607      0.12440     -0.02429 : 12     1    -1
                 0.01588      0.05925     -0.11121 : 11     1     1
                 0.01607      0.09994      0.07797 : 12     1     1
STATE 45:  20383.34
                 0.94760      0.01256      0.97337 : 9     0     0
                 0.02272     -0.03488     -0.14664 : 12     1    -1
                 0.02272      0.03108     -0.14750 : 12     1     1
STATE 46:  20477.11
                 0.98482     -0.86083      0.49376 : 10     0     0
STATE 47:  21050.58
                 0.99056      0.95789     -0.27020 : 11     0     0
STATE 48:  22770.70
                 0.88221     -0.22705     -0.91141 : 13     1     0
                 0.03542      0.05434     -0.18019 : 13     1    -1
                 0.02120      0.07174      0.12672 : 14     1    -1
                 0.03542      0.13252      0.13363 : 13     1     1
                 0.02120      0.00391     -0.14556 : 14     1     1
STATE 49:  22771.43
                 0.03515      0.06002      0.17762 : 13     1     0
                 0.03797     -0.06239     -0.18461 : 14     1     0
                 0.46159     -0.38210     -0.56177 : 13     1    -1
                 0.46159      0.03700      0.67839 : 13     1     1
STATE 50:  22784.29
                 0.03736     -0.07019     -0.18011 : 13     1     0
                 0.48003     -0.57006      0.39379 : 13     1    -1
                 0.48003     -0.68619      0.09578 : 13     1     1
```

Supplementary Table 6. Excited state energies S_i/T_j (bold), singlet-triplet energy differences and SOCCs in cm^{-1} for the ten lowest singlet and triplet states from TDDFT calculations.

		**T_1**		**T_2**		**T_3**		**T_4**		**T_5**	
	E	SOCC	Δ*E*	SOCC	Δ*E*	SOCC	Δ*E*	SOCC	Δ*E*	SOCC	Δ*E*
**S_0**		3.2	**6067**	2.2	**9929**	38.6	**11701**	31.1	**11896**	28.6	**13104**
**S_1**	**13659**	73.0	7591	40.8	7591	29.7	3729	92.5	1958	34.6	1763
**S_2**	**13669**	71.2	7602	39.9	3740	90.2	3740	32.8	1968	13.5	1773
**S_3**	**15108**	30.1	9041	78.4	5179	35.2	3407	57.6	3407	0.9	3212
**S_4**	**15614**	16.5	9547	115.5	5685	69.3	3913	1.4	3718	107.2	3718
**S_5**	**17642**	1.8	11575	1.0	7713	99.3	5941	106.1	5746	20.2	4538
**S_6**	**18168**	0.6	12101	1.2	8239	56.9	6467	31.4	6272	109.8	5064
**S_7**	**18949**	10.7	12882	25.6	9020	41.8	7248	48.0	7053	17.3	5845
**S_8**	**19045**	22.2	12977	21.5	9116	15.5	7344	49.1	7149	50.0	5941
**S_9**	**20346**	2.4	14278	2.4	10416	4.2	8645	6.6	8450	1.0	7242
**S_{10}**	**20451**	2.9	14383	8.5	10521	15.5	8750	14.3	8555	1.4	7347
		**T_6**		**T_7**		**T_8**		**T_9**		**T_{10}**	
	E	SOCC	Δ*E*	SOCC	Δ*E*	SOCC	Δ*E*	SOCC	Δ*E*	SOCC	Δ*E*
**S_0**		103.6	**14623**	0.4	**15815**	92.3	**17156**	91.3	**17377**	1.7	**19377**
**S_1**	**13659**	32.0	965	39.4	2156	9.5	3498	4.2	3718	5.5	5718
**S_2**	**13669**	11.9	954	15.8	2146	28.4	3487	10.8	3708	2.5	5708
**S_3**	**15108**	97.8	485	40.2	707	0.3	2048	0.4	2268	3.4	4269
**S_4**	**15614**	0.6	991	8.9	201	6.1	1542	26.7	1763	1.8	3763
**S_5**	**17642**	25.4	3019	1.8	1828	12.1	486	49.1	266	0.5	1735
**S_6**	**18168**	49.1	3545	0.6	2353	69.0	1012	61.7	792	1.0	1209
**S_7**	**18949**	24.8	3134	98.2	3134	21.1	1793	88.9	1573	13.2	428
**S_8**	**19045**	16.7	3230	96.3	1889	92.9	1889	20.3	1668	5.2	332
**S_9**	**20346**	2.3	4531	7.4	3189	22.8	2969	9.3	2969	88.7	969
**S_{10}**	**20451**	7.0	4636	20.2	3294	1.4	3074	45.3	1074	88.5	1074

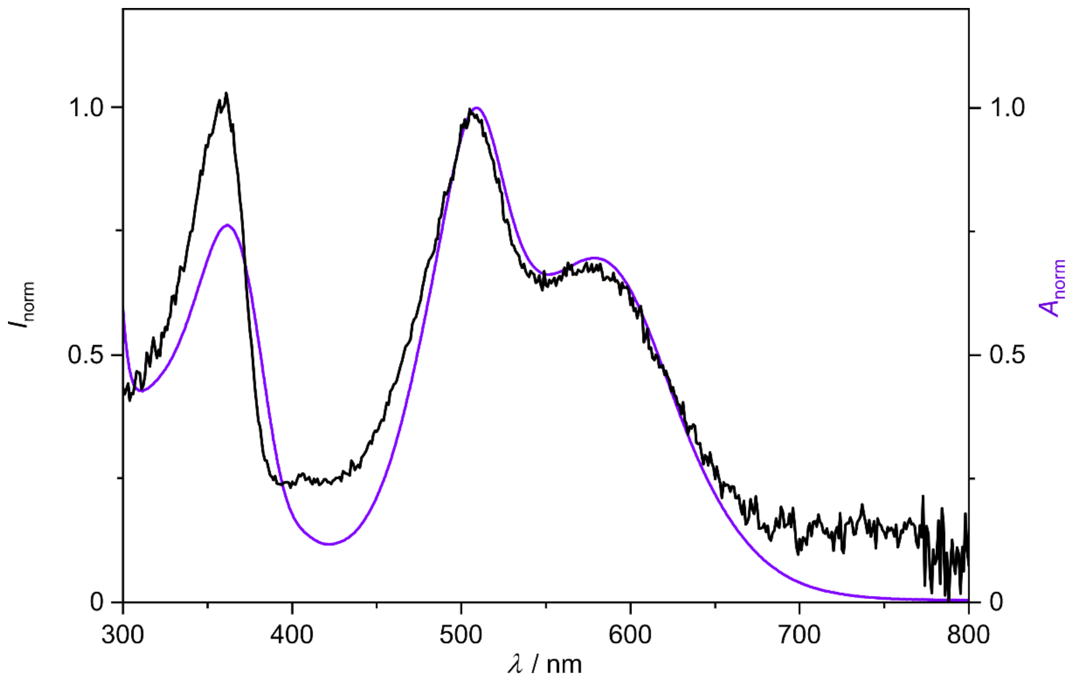

Supplementary Figure 10. Normalized excitation spectrum (black) of [Mn(pbmi)$_2$][OTf] in 2-MeTHF at 77 K observed at 980 nm. Normalized absorption spectrum (purple) of [Mn(pbmi)$_2$][OTf] in 2-MeTHF at 293 K for reference.

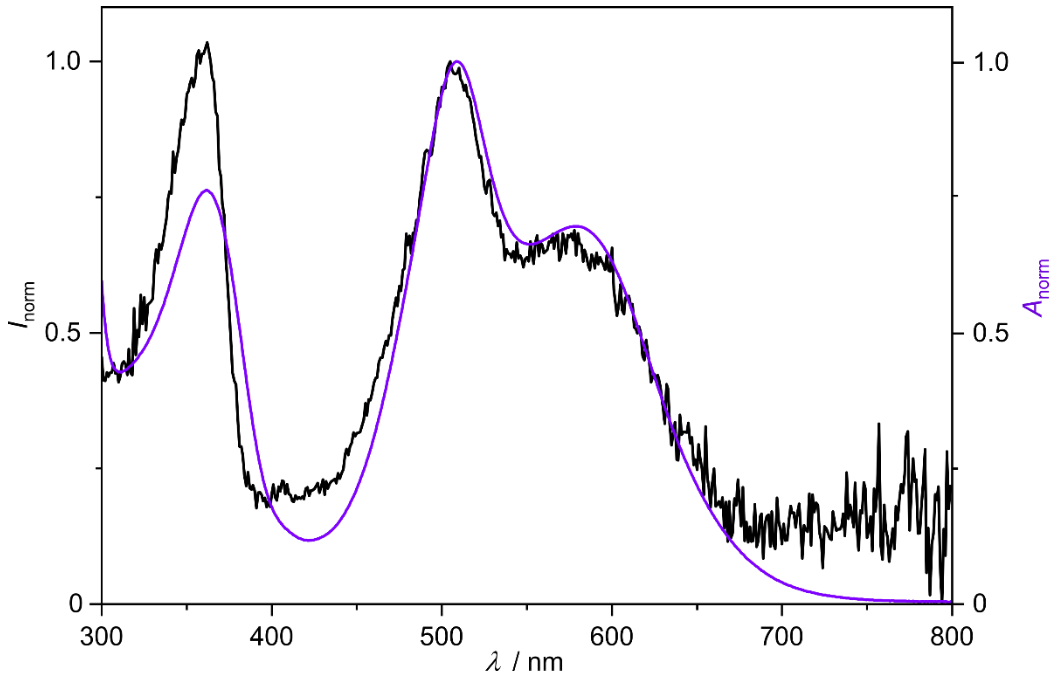

Supplementary Figure 11. Normalized excitation spectrum (black) of [Mn(pbmi)$_2$][OTf] in 2-MeTHF at 77 K observed at 1020 nm. Normalized absorption spectrum (purple) of [Mn(pbmi)$_2$][OTf] in 2-MeTHF at 293 K for reference.

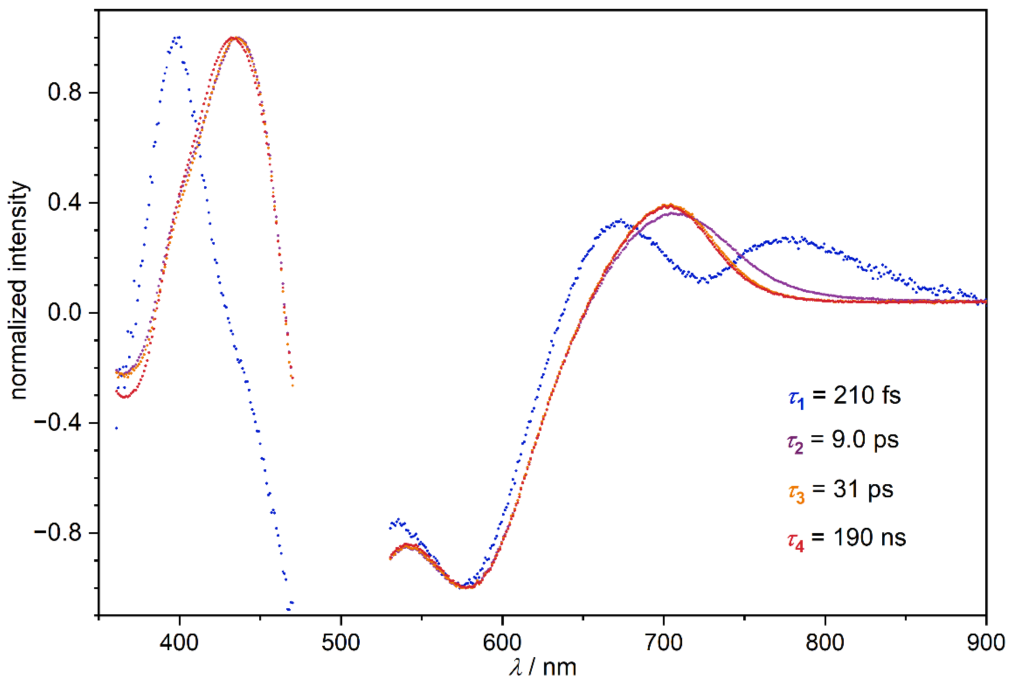

Supplementary Figure 12. Evolution associated difference spectra (EADS) obtained from the transient absorption spectra of [Mn(pbmi)$_2$][OTf] after global analysis in CH_3CN at 293 K after excitation at 505 nm.

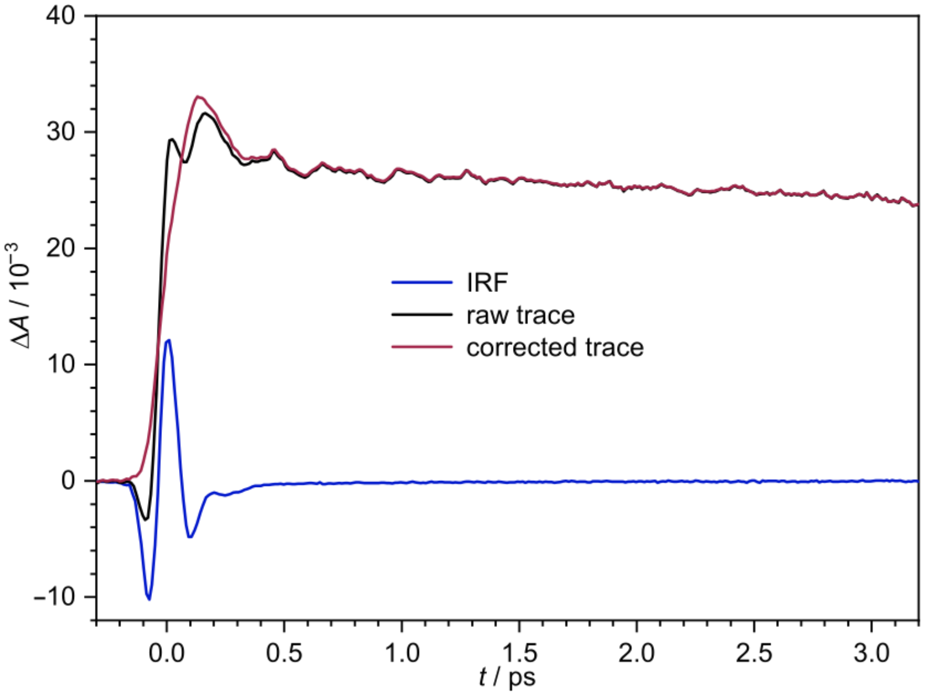

Supplementary Figure 13. Exemplary trace correction at λ = 750 nm from the ultrafast fs transient absorption spectroscopy experiment after excitation of [Mn(pbmi)$_2$][OTf] (280 μM) at 505 nm in CH_3CN at 293 K.

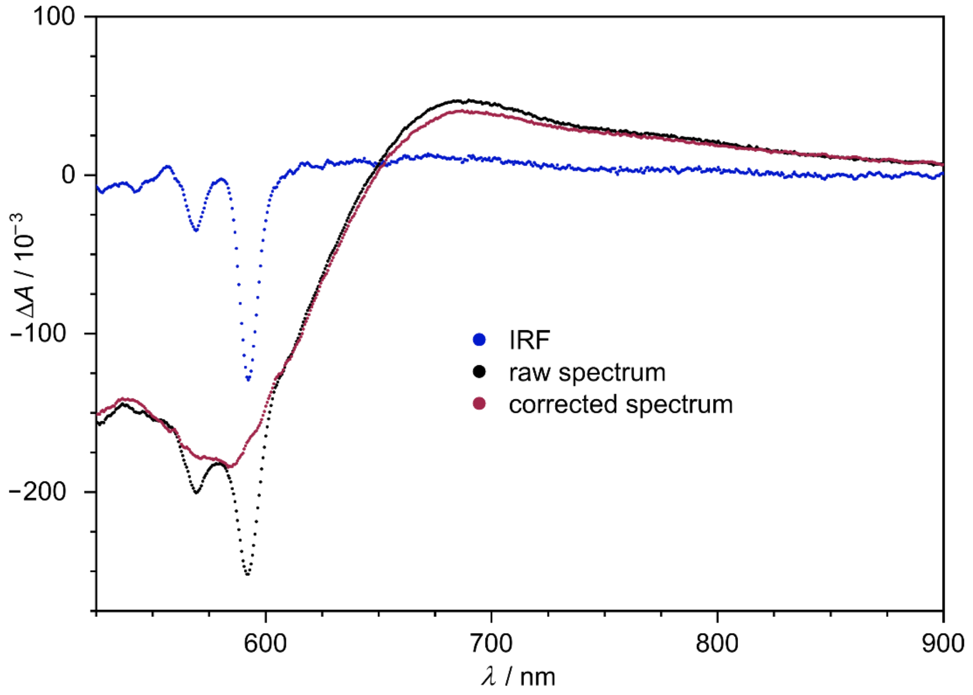

Supplementary Figure 14. Exemplary spectral correction of the ultrafast fs transient absorption spectroscopy experiment in CH_3CN at 293°K at 50 fs.

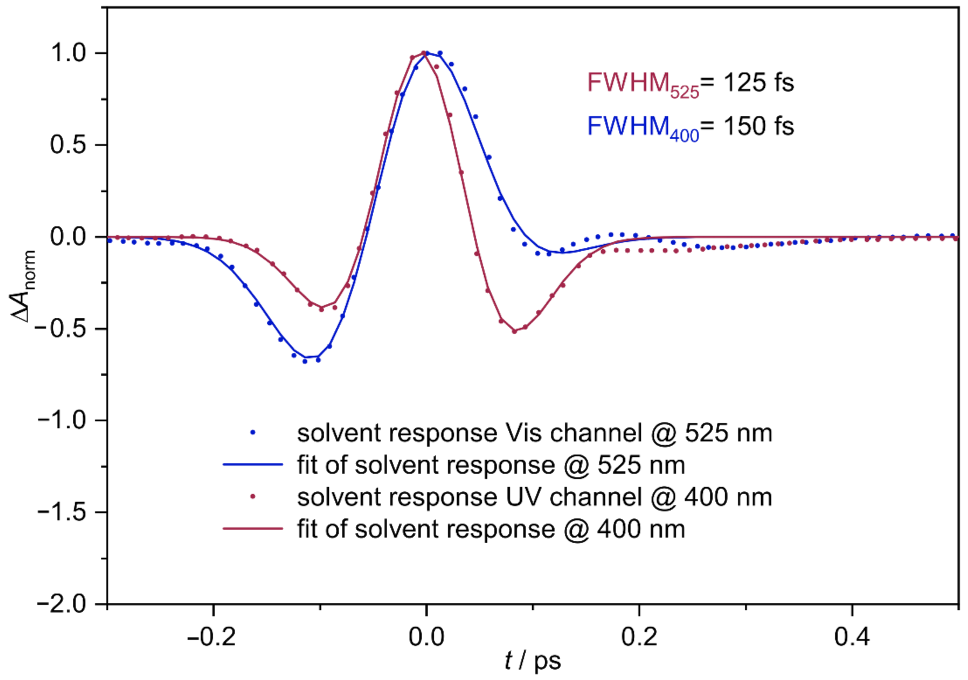

Supplementary Figure 15. Solvent response at $\lambda = 525$ nm and $\lambda = 400$ nm from the ultrafast fs transient absorption spectroscopy experiment in CH_3CN at 293 K.

Supplementary Table 7. Selected DFT calculated molecular orbitals of the [1]GS of $[Mn(pbmi)_2]^+$ with energies given in eV, displayed at an isosurface value of 0.05 a.u.. Hydrogen atoms are omitted for clarity.

LUMO+20 / +1.9753	LUMO+19 / +1.9727	LUMO+18 / +1.9567
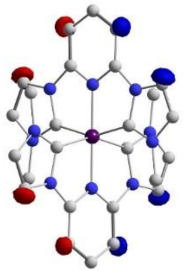	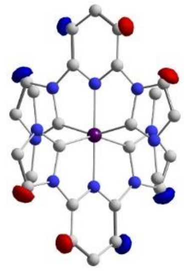	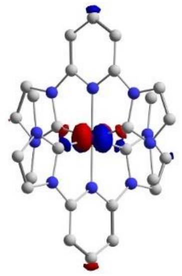
LUMO+17 / +1.7760	LUMO+16 / +1.7363	LUMO+15 / +1.7231
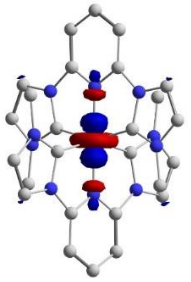	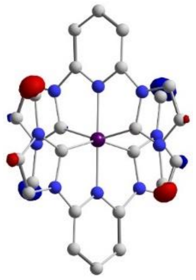	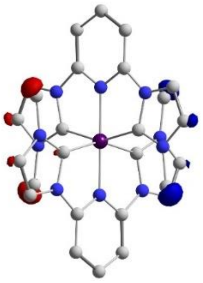
LUMO+14/ +1.6468	LUMO+13 / +1.4984	LUMO+12 / +1.4329
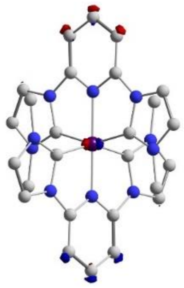	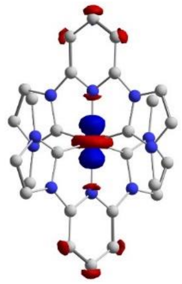	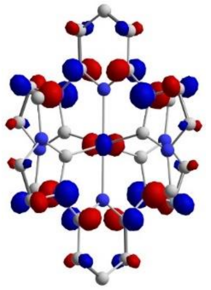
LUMO+11 / +1.2015	LUMO+10 / +0.8535	LUMO+9 / +0.8444
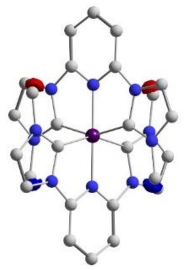	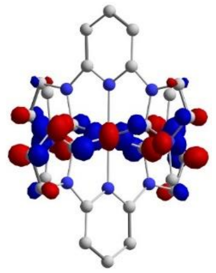	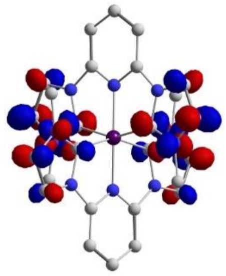
LUMO+8 / +0.6701	LUMO+7 / +0.6659	LUMO+6 / +0.5263
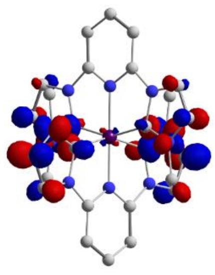	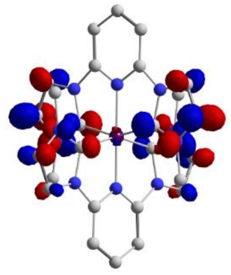	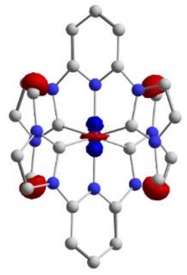

LUMO+5 / +0.4160	LUMO+4 / +0.4094	LUMO+3 / –0.8069
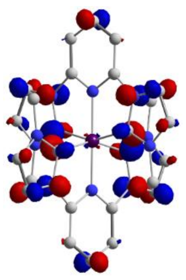	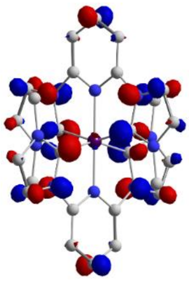	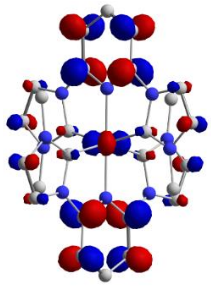
LUMO+2 / –0.9094	LUMO+1 / –1.0454	LUMO / –1.0518
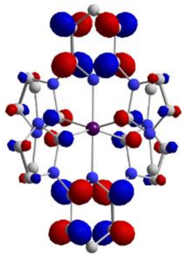	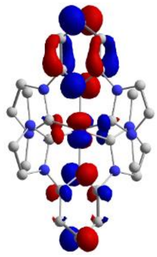	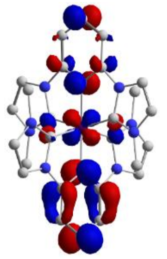
HOMO / –4.1826	HOMO–1 / –4.2584	HOMO–2 / –4.2634
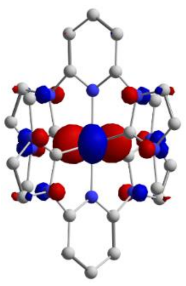	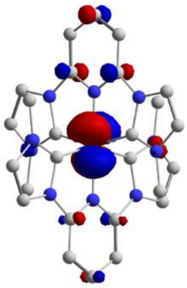	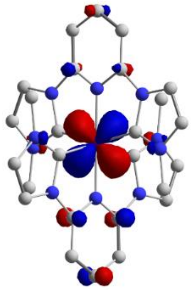

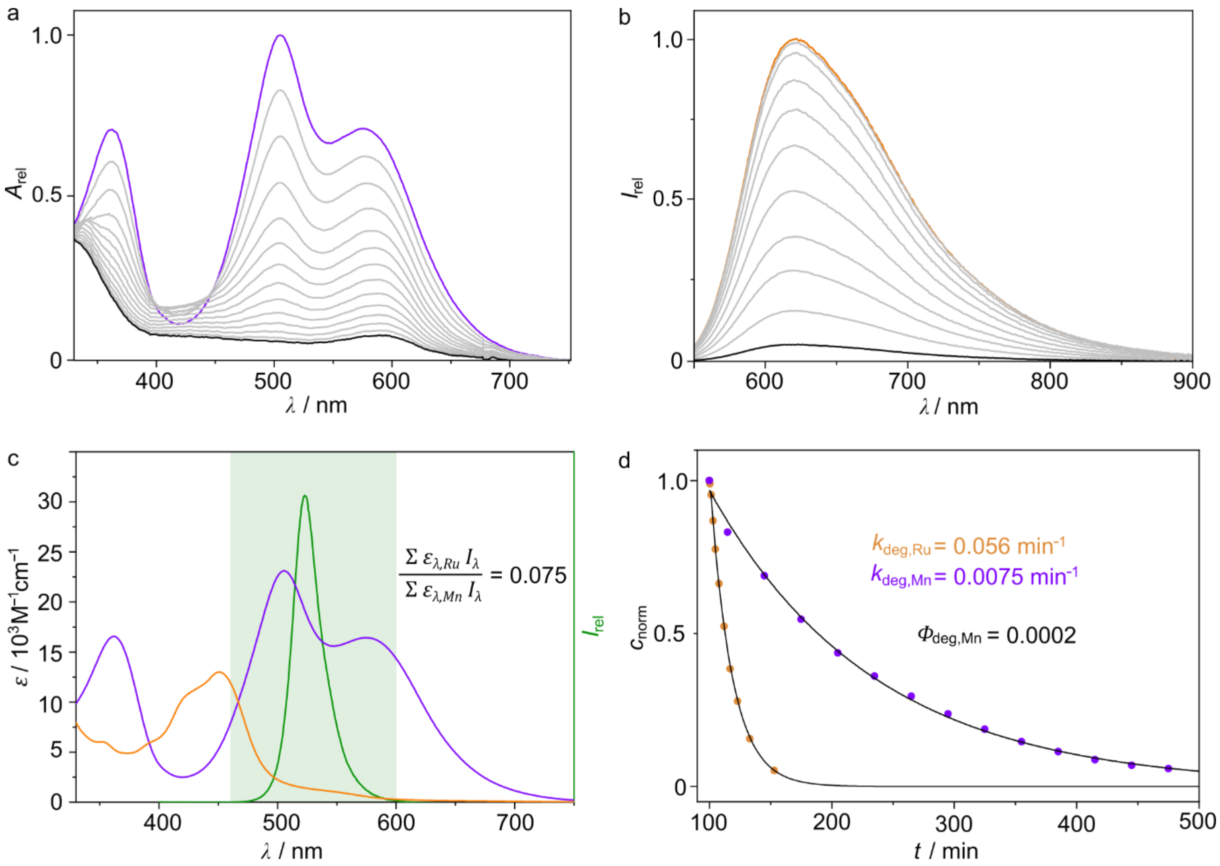

Supplementary Figure 16. a, Absorption spectra before (purple) and after (black) LED irradiation (λ = 523 nm) of a [Mn(pbmi)$_2$][OTf] solution in deaerated CH_3CN at 293 K. **b,** Emission spectra before (orange) and after (black) LED irradiation (λ = 523 nm) of a [Ru(bpy)$_3$][PF$_6$]$_2$ solution in deaerated CH_3CN at 293 K. **c,** Calibrated absorption spectra of [Mn(pbmi)$_2$][OTf] (purple) and [Ru(bpy)$_3$][PF$_6$]$_2$ (orange) in CH_3CN and the emission spectrum of the UHP-LED employed for irradiation (green). The green box shows the wavelength range which has been used for the determination of the weighing factor. **d,** Decay traces of [Mn(pbmi)$_2$][OTf] (purple dots, $\lambda_{obs,abs}$ = 505 nm) and [Ru(bpy)$_3$][PF$_6$]$_2$ (orange dots, $\lambda_{obs,emis}$ = 620 nm) superimposed with the corresponding monoexponential fits of the form $c_0 e^{-k_{deg,Mn} t} + n$ and $c_0 e^{-k_{deg,Ru} t}$, respectively.

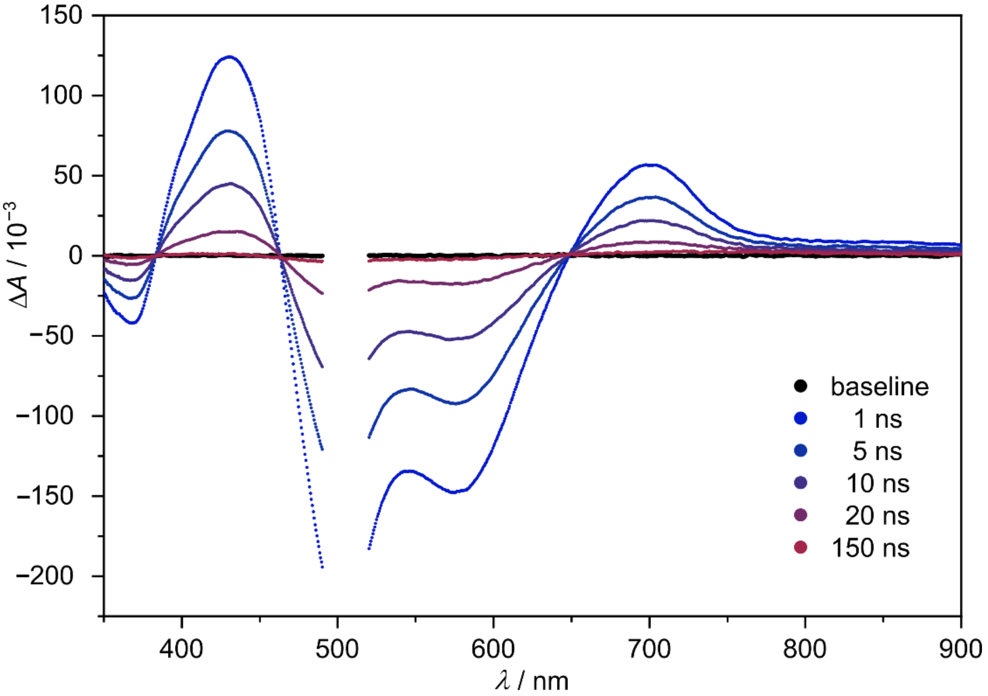

Supplementary Figure 17. Nanosecond transient absorption spectra of [Mn(pbmi)$_2$][OTf] (280 µM) with benzophenone (100 mM) in CH_3CN at 293 K after excitation at 505 nm.

References

1. Baryshnikov, G., Minaev, B., Ågren, H. Theory and Calculation of the Phosphorescence Phenomenon. *Chem. Rev.* **117**, 6500–6537 (2017).
2. Minaev, B., Baryshnikov, G., Agren, H. Principles of phosphorescent organic light emitting devices. *Phys. Chem. Chem. Phys.* **16**, 1719–1758 (2014).
3. Strickler, S. J., Berg, R. A. Relationship between Absorption Intensity and Fluorescence Lifetime of Molecules. *J. Chem. Phys.* **37**, 814–822 (1962).
